# Influence of higher body mass index on postoperative nausea and vomiting in patients following thoracic surgery for lung cancer: a propensity score-matched cohort study

**DOI:** 10.1038/s41598-024-64686-1

**Published:** 2024-06-16

**Authors:** Nan Zhang, Di Feng, Wei Wu, Ji Liu, Hong Shi

**Affiliations:** Department of Anesthesiology, Shanghai Pulmonary Hospital, School of Medicine, Tongji University, Shanghai, 200433 China

**Keywords:** Body mass index, Postoperative nausea and vomiting, Thoracic surgery, Lung cancer, Pain, Cytokine, Medical research, Oncology, Risk factors, Signs and symptoms

## Abstract

This study aimed to quantify the association between body mass index (BMI) and postoperative nausea and vomiting (PONV) within the initial 48 h following thoracic surgery for lung cancer. We then explored whether changes in serum inflammatory factor concentrations were related to BMI during the early postoperative period. We conducted a propensity score-matched (PSM), retrospective cohort study at a specialized tertiary medical center. A total of 194 patients aged 18–80 years who underwent thoracic surgery for lung cancer at Shanghai Pulmonary Hospital between January and June 2021 were enrolled. The primary outcome was the incidence of PONV during the first 48 h after surgery. Nausea, vomiting or retching at different time periods, severe pain, and concentrations of perioperative serum inflammatory factors including CRP, IL-6, IL-12, and IFN-γ were also assessed. Patients in the high BMI group (BMI ≥ 25 kg/m^2^) had a lower incidence of PONV than those in the normal BMI group (18.5–25 kg/m^2^) within the first 48 h after surgery (22 vs. 50%, *p* = 0.004). The incidence of nausea was lower at 0–12 h (14.5 vs. 37.1%, *p* = 0.004) and 12–24 h (8.1 vs. 22.6%, *p* = 0.025) in the high BMI group after surgery, and the incidence of vomiting was lower at 0–12 h (12.9 vs. 30.6%, *p* = 0.017) in higher BMI after surgery. We found no significant difference in the incidence of severe pain [severe static pain (*p* = 0.697) and severe dynamic pain (*p* = 0.158)]. Moreover, higher concentrations of IL-12 (2.24 ± 2.67 pg/ml vs. 1.48 ± 1.14 pg/ml, *p* = 0.048) and IFN-γ [1.55 (1.00) pg/ml vs. 1.30 (0.89) pg/ml, *p* = 0.041] were observed in patients with normal BMI on the first day after surgery. Given this finding, patients with a normal BMI should receive more attention for the prevention of PONV than those with a high BMI following thoracic surgery for lung cancer.

Trial registration: http://www.chictr.org.cn and ChiCTR2100052380 (24/10/2021).

## Introduction

With the increasing number of patients with a high body mass index (BMI) worldwide, more attention is being focused on the clinical outcomes of patients with different BMI during the perioperative period^1,2^. The past few decades have witnessed the rapid development of noncardiac thoracic surgery and enhanced recovery after surgery (ERAS)^3,4^. However, early postoperative clinical outcomes, including postoperative nausea and vomiting (PONV), severe pain, delirium, and dizziness, lead to patient dissatisfaction and delayed hospital discharge^5–8^. Previous studies have shown a protective effect of a high BMI on the inflammatory response^9,10^. Early postoperative clinical outcomes are closely related to inflammatory response, and postoperative serum C-reactive protein (CRP), IL-6, IL-12, and IFN-γ levels can reflect the postoperative inflammatory status of patients. Studies have shown that the application of exogenous IL-12 in patients with cancer can stimulate IFN-γ production, along with an increased incidence of nausea and vomiting^11,12^.

It is estimated that there are 2.2 million new cases and 1.79 million deaths each year, lung cancer is one of the most commonly diagnosed cancers and the leading cause of cancer-related deaths worldwide^13^. Surgery is the primary treatment for patients with early stage lung cancer. With the development of minimally invasive technology, thoracoscopic surgery has become the most common surgical method in thoracic surgery because of its advantages of less trauma, faster recovery, less postoperative pain, and improved quality of life compared with thoracotomy. However, PONV remains an unsolved problem that can increase medical costs and affect the postoperative recovery. Studies have pointed out that the incidence of PONV within 48 h after thoracoscopic examination is 68.42–73.68%, and 57.89% of patients require drug intervention^14^. This study aimed to evaluate whether PONV following thoracic surgery for lung cancer correlates with different BMI values, and to explore whether serum inflammatory factors play a role in the early recovery of patients with different BMI.

## Methods

### Ethical approval/informed consent

This study was approved and the requirement for written informed consent was waived by the Medical Ethics Committee of Shanghai Pulmonary Hospital (#L21-307). This study conformed to the principles outlined in the Declaration of Helsinki.

### Study participants

This retrospective cohort analysis included all patients aged 18–80 years who underwent thoracic surgery for lung cancer between January 2021 and June 2021 at Shanghai Pulmonary Hospital. This study was approved by the Shanghai Pulmonary Hospital Medical Ethics Committee, which waived the requirement for written informed consent. Patients were excluded if they underwent emergency surgery, had chronic pain, or had missing clinical information (e.g., BMI and postoperative pain scores). This study adhered to the STROBE guidelines.

### Exposure and outcomes

Based on the World Health Organization classification, we defined high BMI as a preoperative BMI ≥ 25 kg/m^2^ and normal BMI as 18.5–25 kg/m^2^. Baseline variables, postoperative follow-up data, and laboratory test results were collected from the electronic medical records system of Shanghai Pulmonary Hospital. The primary outcome was the incidence of PONV during the first 48 h after surgery, defined as any episode of nausea, vomiting, or retching^15^. Nausea is a feeling of upset and discomfort in the stomach, which usually leads to an urge to vomit. Vomiting is the forced discharge of stomach contents into the mouth. Retching is an attempt to vomit, usually accompanied by dry heaves and gagging, spasmodic, and rhythmic contraction of the ventilator, without discharging stomach contents.

The secondary outcomes included nausea, retching, or vomiting during 0–12 h, 12–24 h, 24–36 h and 36–48 h after surgery using the simplified PONV impact scale by Myles et al.^16^ (Consisting of two questions: Q1. Have you vomited or retched? Q2. Have you experienced nausea? If yes, has your feeling of nausea interfering with activities of daily living, such as being able to get out of bed, being able to move freely in bed, being able to walk normally, or eating and drinking?), use of rescue antiemetics, incidence of severe pain, incidence of dizziness, and concentrations of related inflammatory factors, including CRP, IL-6, IL-12, and IFN-γ, on the first and second days after surgery (routine detection of inflammatory factors in this institution). Postoperative pain was evaluated using the visual analog scale (VAS), with scores ranging from 0 (no pain) to 10 (worst pain imaginable). Severe pain, which included severe static (at rest) and dynamic (at movement) pain, was defined as a VAS score of ≥ 7 points during the postoperative period within 48 h. The incidence of dizziness was defined as dizziness during the postoperative any time within 48 h.

### Statistical analysis

Continuous variables conforming to normal distribution were presented as mean ± standard deviation and were analyzed using Student’s *t*-test. Continuous variables with non-normal distribution were expressed as median (interquartile range [IQR]) and analyzed using the Mann–Whitney U test. Categorical data were expressed as frequencies, and percentages were compared using the chi-square test.

Propensity score matching (PSM) was used to minimize the potential bias between 2 groups. A propensity score was calculated by logistic regression model based on 5 variables: sex, age, ASA, Charlson index and surgery type. A caliper of 0.05 was selected and the nearest neighboring method was adopted to match the high BMI group and the normal group with a 1:1 ratio using SPSS23.

A multivariate logistic regression analysis was used to test the association between BMI and PONV. We corrected the models using the following potential confounding variables: sex, age (≤ 40 and > 40 years), history of smoking, Charlson index, surgery type, history of surgery, mode of resection, and duration of anesthesia. Based on the results of multivariate logistic regression, receiver operating characteristic (ROC) curves and the corresponding area under the curve (AUC) were calculated for BMI and smoking history. All statistical analyses were performed using SPSS version 23 (IBM, Chicago, IL, USA) and R-studio 3.5.2, with significance defined as a two-tailed *P* < 0.05.

## Results

**Figure 1:**
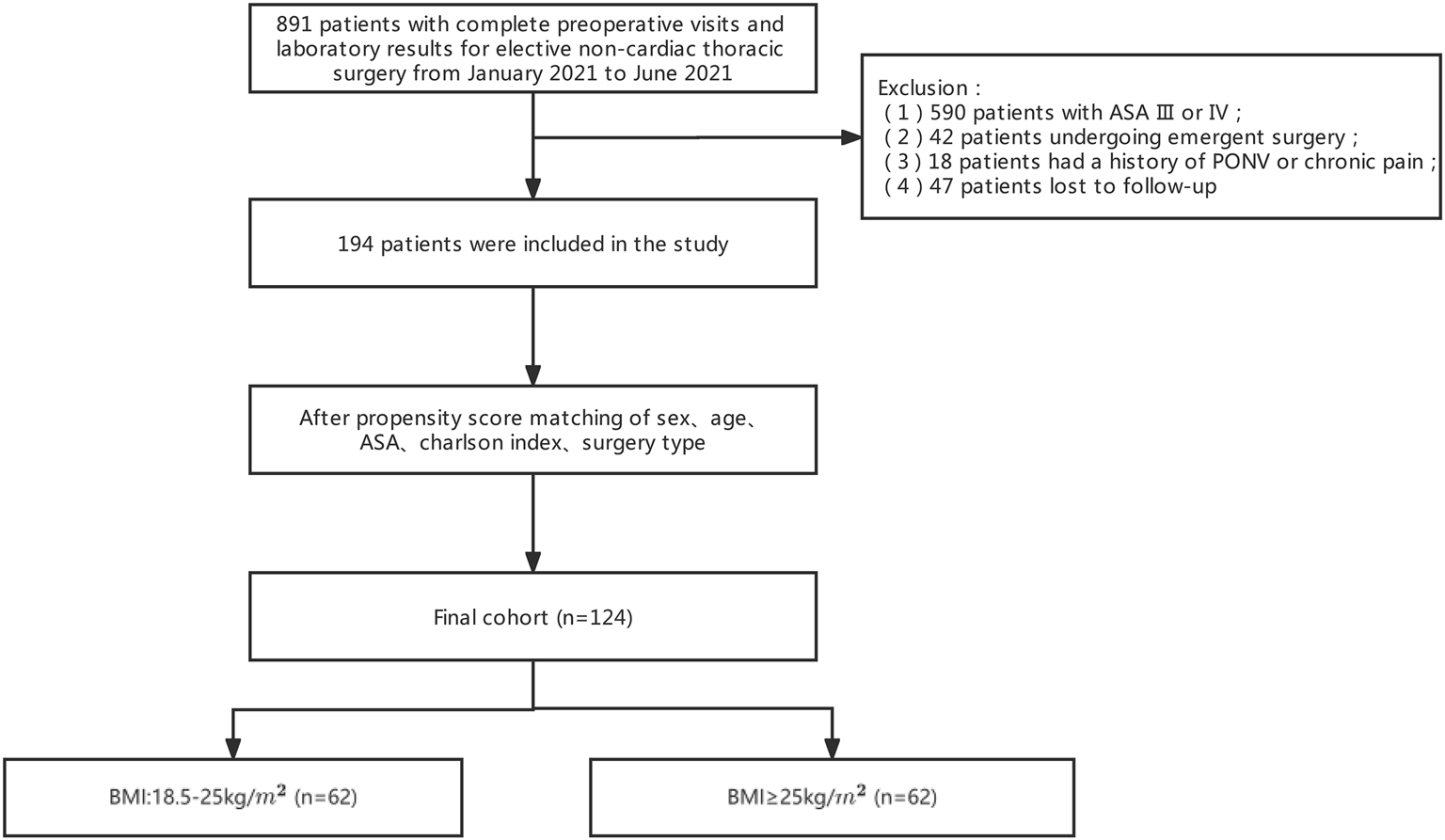
Types and numbers of excluded eligible patients aged between 18 and 80 years who had lung cancer surgeries at Shanghai Pulmonary Hospital between January 2021 and June 2021.

This study enrolled 891 patients aged 18–80 years who underwent thoracic surgery for lung cancer between January 2021 and June 2021 at Shanghai Pulmonary Hospital. After exclusion, 194 patients were eligible for the data analysis. We analyzed the baseline demographic and clinical characteristics using PSM. Before PSM, sex (*P* = 0.041) and ASA grade (*P* = 0.011) differed significantly between the two groups. After PSM for sex, age, ASA, age-adjusted Charlson comorbidity index (aCCI), and surgery type, 62 patients were included in the normal BMI group, which matched the 62 patients in the high BMI group (Fig. 1). The two groups were well-balanced for all baseline demographics after PSM (Table 1). The high BMI group had a median BMI of 26.57 kg/m^2^ with an interquartile range (IQR) of 1.97, whereas the normal BMI group had a median BMI of 22.56 kg/m^2^ with an IQR of 2.61. Almost all the patients underwent VATS, and there was no difference in the Apfel PONV risk score between the two groups before surgery. The duration of anesthesia for all patients was maintained within 3 h. Supplementary Table 1 provides the intraoperative data with details on the intraoperative anesthetic data.

**Table 1:** Baseline characteristics after propensity score matching.

Characteristic	BMI: 18.5–25 kg/m^2^	BMI ≥ 25 kg/m^2^	*P*-value
Number of patients (n)	62	62	
Age [years; mean (SD)]	57.87 ± 13.63	59.93 ± 9.98	0.440
Sex, male [n (%)]	33 (53.2)	34 (54.8)	0.857
Surgery type			
Open	3 (4.8)	3 (4.8)	1.0
VATS	59 (95.2)	59 (95.2)	
ASA-physical status			0.857
I	32 (51.6)	31 (50.0)	
II	30 (48.4)	31 (50.0)	
aCCI	3.40 ± 1.49	3.52 ± 1.42	0.667
Apfel PONV risk score			0.992
1	14(22.6)	13(21.0)	
2	18(29.0)	21(33.9)	
3	27(43.5)	26(41.9)	
4	3(4.9)	2(3.2)	
Resection mode			0.934
Wedge resection	15 (24.2)	16 (25.8)	
Segmental resection	15 (24.2)	17 (27.4)	
Lobectomy	29 (46.8)	27 (43.5)	
Other	3 (4.8)	2 (3.2)	
Duration of anaesthesia; min	112.06 ± 62.52	100.56 ± 48.92	0.256
previous history of surgery	33 (53.2)	35 (56.5)	0.718

Continuous variables are presented as mean ± standard deviation. Categorical variables were presented as numbers (percentages). Abbreviations: aCCI, age-adjusted Charlson comorbidity index; SD, standard deviation; VATS, video-assisted thoracoscopic surgery; ASA, American Society of Anesthesiologists; BMI, body mass index; PONV, postoperative nausea and vomiting.

The higher BMI group had a lower incidence of PONV within 48 h after surgery (22% vs. 50%, *p* = 0.004). In addition, 43.5% of the 62 patients in the normal BMI group and 14.5% of the patients in the higher BMI group experienced nausea (*p* ≤ 0.001). The normal BMI group had a higher incidence of vomiting or retching (32.3% vs. 12.9%, *p* = 0.01). 37.1% in the normal BMI group and 14.5% in the higher BMI group of patients experienced nausea episodes within 12 h after surgery (*p* = 0.004), while 22.6% in the normal BMI group and 8.1% in the higher BMI group of patients experienced nausea episodes within 12–24 h after surgery (*p* = 0.025). 30.6% in the normal BMI group and 12.9% in higher BMI group of the patients experienced vomiting or retching episodes within 12 h after surgery (*p* = 0.017). There was no difference in the incidence of nausea, vomiting, or retching at other time points and in the use of rescue antiemetics.

There was no significant difference in the incidence of severe pain (severe static pain (*p* = 0.697) and severe dynamic pain (*p* = 0.158)). However, no significant difference was observed in the incidence of dizziness between the two groups (*p* = 0.571). Table 2 summarizes the data on postoperative clinical outcomes within the initial postoperative 48 h.

**Table 2:** Postoperative clinical outcomes.

	BMI: 18.5–25 kg/m^2^	BMI ≥ 25 kg/m^2^	*P*-value
Primary outcome			
PONV 0–48 h after surgery	31 (50.0%)	9 (22.0%)	0.004
Secondary outcome			
Nausea			
0–12 h	23 (37.1%)	9 (14.5%)	0.004
12–24 h	14 (22.6%)	5 (8.1%)	0.025
24–36 h	4 (6.5%)	0 (0)	0.042
36–48 h	3 (4.8%)	0 (0)	0.08
Nausea 0–48 h after surgery	27 (43.5%)	9 (14.5%)	0.000
Vomiting/retching			
0–12 h	19 (30.6%)	8 (12.9%)	0.017
12–24 h	4 (6.5%)	4 (6.5%)	1.00
24–36 h	1 (1.6%)	0 (0)	0.315
36–48 h	0 (0)	0 (0)	1
Vomiting/retching 0–48 h after surgery	20 (32.3%)	8 (12.9%)	0.01
Rescue antiemetic	8 (12.9%)	4 (6.5%)	0.224
VAS-static (%)	4 (6.5%)	3 (4.8%)	0.697
VAS-dynamic (%)	14 (22.6%)	8 (12.9%)	0.158
Dizziness	23 (37.1%)	20 (32.3%)	0.571

Categorical variables are expressed as number (percentage).

VAS-static, VAS score for severe dynamic pain; VAS-dynamic, VAS score for severe dynamic pain; BMI, body mass index.

**Figure 2:**
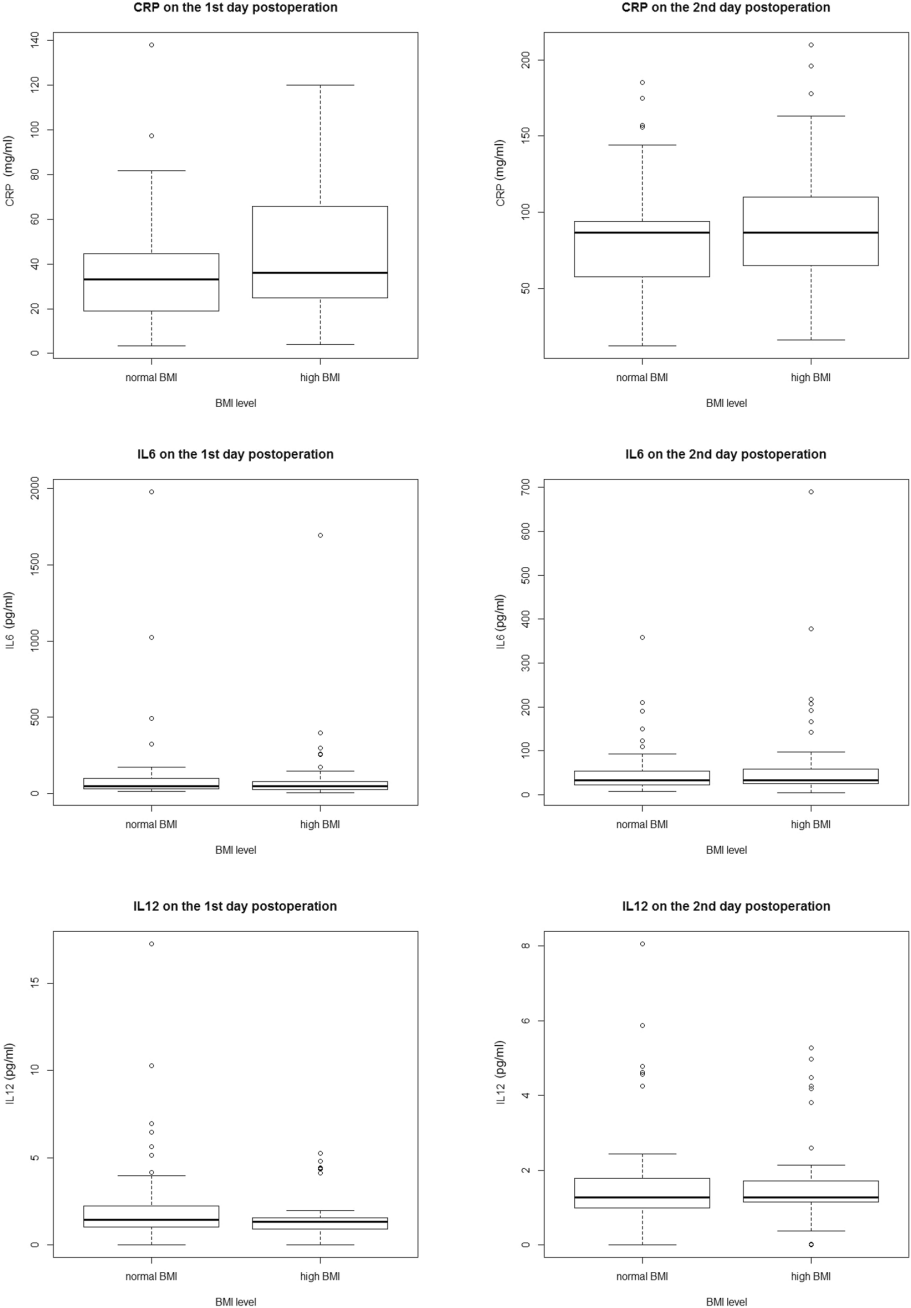

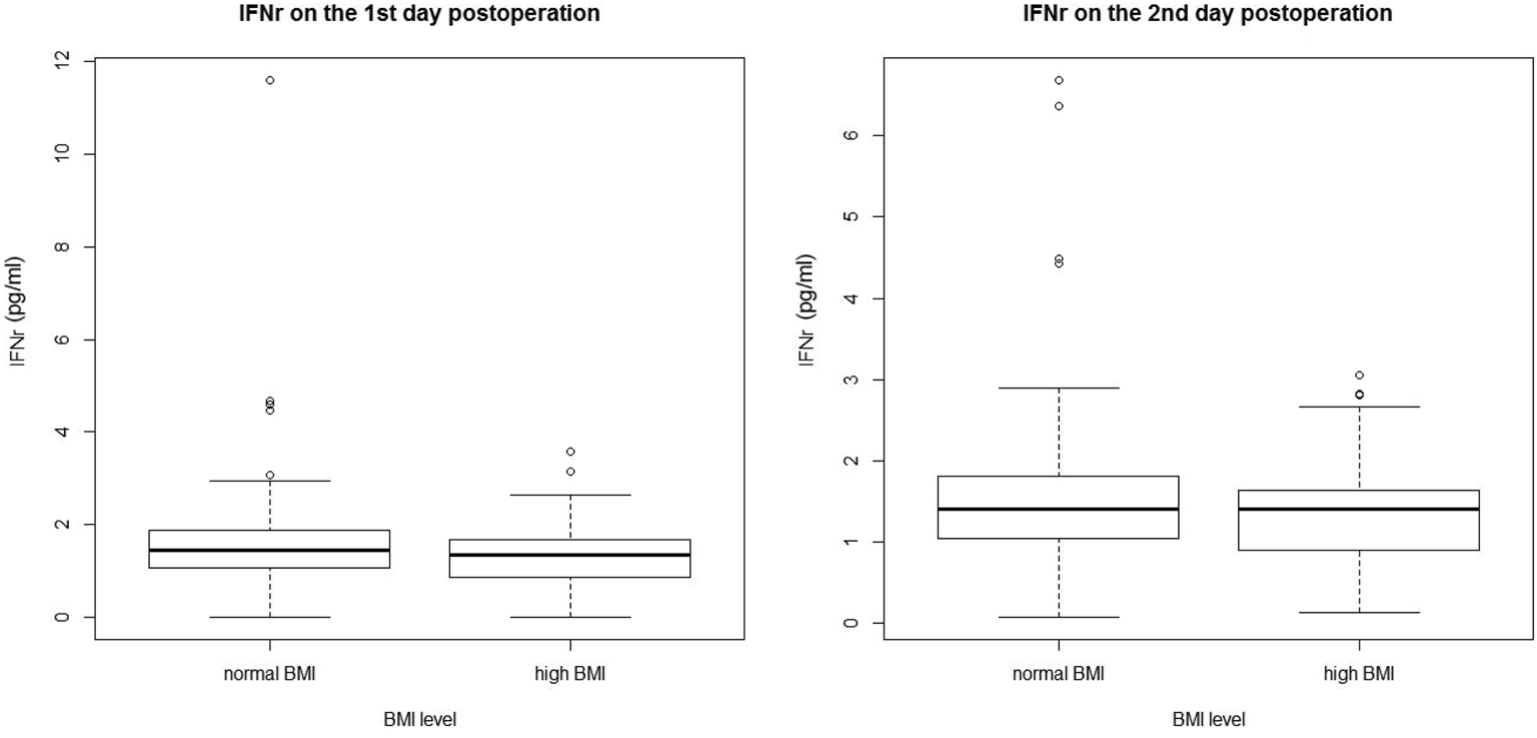
Inflammatory factors in the normal and high BMI groups on the initial 2 days after surgery. Abbreviations: BMI, body mass index; 1st, first; 2nd, second.

The concentrations of postoperative serum inflammatory factors in the two groups within the first postoperative day 2 are plotted in Fig. 2. Notably, on the first day after surgery, the concentrations of IL-12 (2.24 ± 2.67 pg/ml vs. 1.48 ± 1.14 pg/ml, *p* = 0.048) and IFN-γ [1.55(1.00) pg/ml vs. 1.30 (0.89) pg/ml, *p* = 0.041] were higher in the normal BMI group than in the higher BMI group. On the second day, the concentrations of IL-12 and IFN-γ did not differ between the two groups. And there were no significant differences in the baseline concentrations of serum CRP (3.357 ± 0.68 mg/ml vs. 3.417 ± 0.78 mg/ml), IL-6 [3.50 (6.69) pg/ml vs. 4.13 (4.82) pg/ml], IL-12 (2.26 ± 3.10 pg/ml vs. 1.72 ± 1.23 pg/ml), and IFN-γ [1.64 (1.37) pg/ml vs. 1.34 (0.82) pg/ml] between the groups. There was no difference in the CRP and IL-6 levels between the groups on postoperative day 2.

Age, sex, aCCI, duration of anesthesia, surgical history, surgery type, and resection mode were not significantly associated with the incidence of PONV. Smoking history was not significantly associated with the incidence of postoperative nausea; however, the incidence of postoperative vomiting or retching was 79% lower in patients who had smoked previously than in those without a smoking history (OR [odds ratio] = 0.21, 95% CI [0.05–0.95], *p* = 0.042). Additionally, BMI was associated with the incidence of PONV (Table 3). Multivariate logistic regression was conducted by including clinical variables to identify risk factors for PONV (Table 4). The OR for BMI was consistently significant in all three models, irrespective of whether BMI was analyzed as a continuous or categorical variable (nausea: OR = 0.16, *p* < 0.05; vomiting/retching: OR = 0.32, *p* < 0.05). When BMI was evaluated as a continuous variable in the fully adjusted model (model 3) the adjusted OR was 0.8 (95% CI, 0.69–0.94) for nausea and 0.8 (95% CI, 0.67–0.94) for vomiting or retching.

**Table 3:** Results of univariate analysis of PONV.

Variable	Nausea	*p*-value	Vomiting	*p*-value
	OR 95% CI		OR 95% CI	
Age	1.02 (0.98–1.06)	0.270	0.99 (0.96–1.03)	0.684
Sex				
Male	Ref		Ref	
Female	1.52 (0.70–3.33)	0.294	1.85 (0.79–4.34)	0.16
BMI (kg/m^2^)	0.83 (0.72–0.95)	0.009	0.81 (0.7–0.95)	0.01
aCCI	1.12 (0.85–1.48)	0.431	0.9 (0.66–1.22)	0.482
Duration of anaesthesia (min)	1 (0.99–1.01)	0.687	1 (0.99–1.01)	0.499
Smoking status, n (%)				
No	Ref		Ref	
Yes	0.34 (0.11–1.06)	0.062	0.21 (0.05–0.95)	0.042
Resection mode				
Wedge resection of lung	Ref		Ref	
Segmental resection of lung	1.98 (0.65–6.06)	0.229	0.92 (0.28–3.03)	0.887
Lobectomy of lung	1.26 (0.45–3.52)	0.663	0.95 (0.33–2.72)	0.924
Other	0.79 (0.07–8.24)	0.841	0.79 (0.07–8.24)	0.841
Surgery type	0.84 (0.15–4.8)	0.844	1.53 (0.17–13.71)	0.702
History of surgery	1.34 (0.63–3.125)	0.409	1.10 (0.467–2.564)	0.83

PONV, postoperative nausea and vomiting; BMI, body mass index; aCCI, age-adjusted Charlson comorbidity index.

**Table 4:** Multivariate-adjusted ORs and 95% CI for BMI associated with nausea and vomiting.

Variable	Unadjusted		Model 1		Model 2		Model 3	
	OR (95% CI)	*P*-value	OR (95% CI)	*P*-value	OR (95% CI)	*P*-value	OR (95% CI)	*P*-value
Nausea								
BMI (kg/m^2^)	0.83 (0.72–0.95)	0.009	0.81 (0.7–0.94)	0.007	0.81 (0.7–0.94)	0.006	0.8 (0.69–0.94)	0.005
BMI (18.5–25 kg/m^2^)	Ref		1 (Ref)		1 (Ref)		1 (Ref)	
BMI (≥ 25 kg/m^2^)	0.23 (0.1–0.56)	0.001	0.21 (0.09–0.52)	0.001	0.19 (0.08–0.5)	0.001	0.16 (0.06–0.44)	< 0.001
Vomiting/retching								
BMI (kg/m^2^)	0.81 (0.7–0.95)	0.01	0.81 (0.69–0.96)	0.013	0.8 (0.68–0.95)	0.009	0.8 (0.67–0.94)	0.009
BMI (18.5–25 kg/m^2^)			1 (Ref)		1 (Ref)		1 (Ref)	
BMI (≥ 25 kg/m^2^)	0.33 (0.13–0.82)	0.017	0.32 (0.13–0.82)	0.018	0.32 (0.12–0.84)	0.021	0.32 (0.12–0.84)	0.021

Model 1 adjusted for age, sex, and ASA level.

Model 2 was adjusted for model 1 + aCCI, smoking status, and history of surgery.

Model 3 was adjusted for model 2 + surgery type, resection mode, and duration of anesthesia.

aCCI, age-adjusted Charlson comorbidity index; OR, odds ratio; CI, confidence interval; Ref, reference.

According to the results of the multivariate analysis, two variables (BMI and smoking history) were selected to construct an ROC curve (Supplementary Fig. 1). The AUC for predicting nausea was 0.710 (95% CI, 0.611–0.809), with a sensitivity and specificity of 66.7 and 72.7%, respectively. Based on the ROC curves, the AUC for vomiting/retching according to BMI and history of smoking was 0.713 (95% CI, 0.614–0.812), with the best sensitivity and specificity of 64.3% and 68.7%, respectively.

Subgroup analyses were performed to assess the impact of BMI (per 1-unit increment) on PONV in distinct subgroups (Supplementary Fig. 2). Associations between BMI and PONV were coordinated in the following subgroups: postoperative nausea, sex (male vs. female; *p*-interaction = 0.539), smoking status (yes vs. no; *p*-interaction = 0.375), ASA classification (1 vs. 2; *p*-interaction = 0.789), history of surgery (yes vs. no; *p*-interaction = 0.657), postoperative vomiting/retching, sex (male vs. female; *p*-interaction = 0.619), smoking status (yes vs. no; *p*-interaction = 0.362), ASA classification (1 vs. 2; *p*-interaction = 0.978), and history of surgery (yes vs. no; *p*-interaction = 0.756).

## Discussion

PONV is one of the most common postoperative complications, occurring in approximately 30% of surgical patients and more than 70% of high-risk patients^17,18^. Our results demonstrated that patients with normal BMI had a higher incidence of PONV. PONV onset is a dynamic process. We observed the occurrence of PONV during different time periods. We found that postoperative nausea mainly occurred within 24 h after surgery, and postoperative vomiting or retching mainly occurred within 12 h after surgery. In addition, transiently elevated concentrations of IL-12 and IFN-γ were observed in the early postoperative period compared with those with higher BMI.

Currently, there is no consensus on the influence of BMI on PONV. Consistent with our results, some studies have revealed that higher BMI is associated with a lower risk of PONV in the early postoperative period^19–22^. This may be related to the decreased release of histamine and dopamine, neurotransmitters that mediate nausea and vomiting. During perioperative fasting, the amount of hypothalamic histamine in overweight patients is significantly reduced compared with that in patients with normal or lower body weight^23,24^. At the same time, the dopamine receptor level in overweight patients is lower than that in patients with normal BMI^25,26^. In our study, multivariate logistic regression analysis also showed that an increase in BMI reduced the risk of PONV. Univariate logistic regression and ROC curve analysis revealed that the incidence of PONV was lower in patients with a history of smoking than in non-smokers. This finding is consistent with the results of previous studies^27–29^. In Apfel's risk assessment system, the risk factors for PONV include female sex, nonsmoking status, postoperative use of opioids, and history of PONV or motion sickness^30^. In the univariate analysis in our study, non-smoking status was a possible risk factor for PONV. In addition, we successfully constructed a ROC curve for BMI and smoking history to predict PONV. Our results may identify the likelihood of PONV occurrence following thoracic surgery for lung cancer.

Obese patients have been reported to experience more postoperative pain than those with normal BMI^31,32^. However, we found no difference in the incidence of severe postoperative pain between the two groups. This may be related to the most popular surgical modality at our medical center. In our study, most surgeries were uniportal video-assisted thoracic surgeries, wherein a single incision was made in the chest wall. Improvement in the surgical technique is an important part of ERAS, which directly reduces the severity of pain in patients following thoracic surgery. In addition, the BMI of patients in our high BMI group rarely exceeded 30 kg/m^2^, which may explain the similar severe pain scores between the two groups.

There was no difference in CRP and IL-6 concentrations between the two groups. Previous studies have found that PONV changes with postoperative concentrations of CRP and IL-6^33^. However, in the present study, the postoperative serum inflammatory concentrations of CRP and IL-6 showed no difference between the two groups within the first 2 days after surgery. A previous study indicated that IL-12 and IFN-γ levels were increased in people with higher BMI^34,35^; however, in this study, we found that serum IL-12 and IFN-γ levels were higher in patients with a normal BMI than in those with a high BMI on the first day after surgery. Consistent with our study, Huang et al. reported that the improvement in patients’ quality of life (including reduced nausea, vomiting, and pain) was associated with regulation of the inflammatory cascade comprising IL-12 and IFN-γ^33^. IL-12 promotes the production of IFN-γ and induces a Th1 response in various disease models^36–38^. More evidence is needed to determine whether patients with different BMI have different Th1 immunomodulatory mechanisms that affect their quality of life or long-term prognosis after surgery.

Our study had some limitations that warrant consideration. First, it is important to acknowledge that this was a retrospective study, which inherently carries the potential for bias. Second, it is pertinent to note that our study sample comprised individuals of the Chinese ethnicity. Thus, extrapolating our findings to other ethnic groups may require additional research for validation and generalization. Third, the sample size of individuals with BMI < 18 kg/m^2^ and > 30 kg/m^2^ in our study was limited. While the distribution of the population aligns with the demographic profile of China, caution should be exercised when generalizing the findings.

## Conclusions

Patients with a normal BMI were more prone to PONV than those with a higher BMI following thoracic surgery for lung cancer. The elevated concentrations of pro-inflammatory factors, including IL-12 and IFN-γ, in patients with normal BMI indicate that inflammation may be associated with the pathological process of PONV and may affect the prognosis of patients with different BMI.

### Supplementary Information

Supplementary Information.

## Data Availability

The datasets generated and/or analyzed during the current study are available from the corresponding author upon reasonable request.

## Abbreviations

BMI: Body mass index
PSM: Propensity score-matched
PONV: Postoperative nausea and vomiting
ERAS: Enhanced recovery after surgery
CRP: C-reactive protein
ASA: American Society of Anesthesiologists
VAS: Visual analog scale
aCCI: Age-adjusted Charlson comorbidity index
IQR: Interquartile range
ROC: Receiver operating characteristic
AUC: Area under the curve

## References

[1] Hajmohamed S, Patel D, Apruzzese P, Kendall MC, De Oliveira G (2021). Early postoperative outcomes of super morbid obese compared to morbid obese patients after ambulatory surgery under general anesthesia: A propensity-matched analysis of a national database. Anesth. Analg..

[2] Cohen B, Tanios MA, Koyuncu O (2020). Association between higher BMI and postoperative pain and opioid consumption in pediatric inpatients - A retrospective cohort study. J. Clin. Anesth..

[3] Rice D, Rodriguez-Restrepo A, Mena G (2021). Matched pairs comparison of an enhanced recovery pathway versus conventional management on opioid exposure and pain control in patients undergoing lung surgery. Ann. Surg..

[4] Forster C, Doucet V, Perentes JY (2021). Impact of an enhanced recovery after surgery pathway on thoracoscopic lobectomy outcomes in non-small cell lung cancer patients: A propensity score-matched study. Transl. Lung Cancer Res..

[5] Dhawan R, Daubenspeck D, Wroblewski KE (2021). Intrathecal morphine for analgesia in minimally invasive cardiac surgery: A randomized, placebo-controlled. Double-Blinded Clin. Trial. Anesthesiol..

[6] Gao X, Zhao T, Xu G, Ren C, Liu G, Du K (2021). The efficacy and safety of ultrasound-guided, bi-level, erector spinae plane block with different doses of dexmedetomidine for patients undergoing video-assisted thoracic surgery: A randomized controlled trial. Front. Med..

[7] Majumdar JR, Vertosick E, Long M, Cansino C, Assel M, Twersky R (2019). Effects of midazolam on postoperative nausea and vomiting and discharge times in outpatients undergoing cancer-related surgery. AANA J..

[8] Evered LA, Chan MTV, Han R (2021). Anaesthetic depth and delirium after major surgery: A randomised clinical trial. Br. J. Anaesth.

[9] Mica L, Vomela J, Keel M, Trentz O (2014). The impact of body mass index on the development of systemic inflammatory response syndrome and sepsis in patients with polytrauma. Injury.

[10] Mica L, Keller C, Vomela J, Trentz O, Plecko M, Keel MJ (2014). The impact of body mass index and gender on the development of infectious complications in polytrauma patients. Eur. J. Trauma Emergency Surgery: Off. Publicat. Eur. Trauma Soc..

[11] Parihar R, Nadella P, Lewis A (2004). A phase I study of interleukin 12 with trastuzumab in patients with human epidermal growth factor receptor-2-overexpressing malignancies: Analysis of sustained interferon gamma production in a subset of patients. Clin. Cancer Res.: Off. J. Am. Associat. Cancer Res..

[12] Lenzi R, Rosenblum M, Verschraegen C (2002). Phase I study of intraperitoneal recombinant human interleukin 12 in patients with Müllerian carcinoma, gastrointestinal primary malignancies, and mesothelioma. Clin. Cancer Res.: Off. J. Am. Associat. Cancer Res..

[13] Thai AA, Solomon BJ, Sequist LV, Gainor JF, Heist RS (2021). Lung cancer. Lancet (London, England)..

[14] Vijitpavan A, Kittikunakorn N, Komonhirun R (2022). Comparison between intrathecal morphine and intravenous patient control analgesia for pain control after video-assisted thoracoscopic surgery: A pilot randomized controlled study. PloS One.

[15] Apfel CC, Korttila K, Abdalla M (2004). A factorial trial of six interventions for the prevention of postoperative nausea and vomiting. The New Engl. J. Med..

[16] Myles PS, Wengritzky R (2012). Simplified postoperative nausea and vomiting impact scale for audit and post-discharge review. Br. J. Anaesthesia.

[17] Gan TJ (2002). Postoperative nausea and vomiting–can it be eliminated?. Jama.

[18] Frenzel JC, Kee SS, Ensor JE, Riedel BJ, Ruiz JR (2010). Ongoing provision of individual clinician performance data improves practice behavior. Anesthesia Analgesia.

[19] Li TT, Xiong LL, Huang J (2020). The effects of body mass index on the use of patient-controlled intravenous analgesia after open gastrointestinal tumor surgery: A retrospective analysis. J. Pain Res..

[20] Wang Y, Yang Q, Lin J (2020). Risk factors of postoperative nausea and vomiting after total hip arthroplasty or total knee arthroplasty: A retrospective study. Ann. Transl. Med..

[21] Wei H, Gao J, Wang M (2021). Impact of preoperative body mass index on perioperative outcomes is optimized by enhanced recovery protocols in laparoscopic radical cystectomy with intracorporeal urinary diversion. Transl. Androl. Urol..

[22] Kim JH, Hong M, Kim YJ, Lee HS, Kwon YS, Lee JJ (2020). Effect of body mass index on postoperative nausea and vomiting: Propensity analysis. J. Clin. Med..

[23] Boden G, Chen X, Mozzoli M, Ryan I (1996). Effect of fasting on serum leptin in normal human subjects. J. Clin. Endocrinol. metabolism.

[24] Jørgensen EA, Knigge U, Warberg J, Kjaer A (2007). Histamine and the regulation of body weight. Neuroendocrinology.

[25] Wang GJ, Volkow ND, Logan J (2001). Brain dopamine and obesity. Lancet (London, England).

[26] Volkow ND, Wang GJ, Fowler JS, Telang F (2008). Overlapping neuronal circuits in addiction and obesity: Evidence of systems pathology. Philosophical Trans. Royal Soc. London Ser. B, Biol. Sci..

[27] Chatterjee S, Rudra A, Sengupta S (2011). Current concepts in the management of postoperative nausea and vomiting. Anesthesiol. Res. Practice.

[28] Gan TJ, Belani KG, Bergese S (2020). Fourth consensus guidelines for the management of postoperative nausea and vomiting. Anesthesia Analgesia.

[29] Sweeney BP (2002). Why does smoking protect against PONV?. Br. J. Anaesthesia.

[30] Apfel CC, Läärä E, Koivuranta M, Greim CA, Roewer N (1999). A simplified risk score for predicting postoperative nausea and vomiting: Conclusions from cross-validations between two centers. Anesthesiology.

[31] Majchrzak M, Brzecka A, Daroszewski C (2019). Increased pain sensitivity in obese patients after lung cancer surgery. Front. Pharmacol..

[32] Basem JI, White RS, Chen SA (2021). The effect of obesity on pain severity and pain interference. Pain Manag..

[33] Wu Y, Lu X, Ma Y (2018). Perioperative multiple low-dose Dexamethasones improves postoperative clinical outcomes after Total knee arthroplasty. BMC Musculoskeletal Disord..

[34] Schmidt FM, Weschenfelder J, Sander C (2015). Inflammatory cytokines in general and central obesity and modulating effects of physical activity. PloS One.

[35] Iodice S, Ceresa A, Esposito CM (2021). The independent role of body mass index (BMI) and severity of depressive symptoms on biological changes of women affected by overweight/obesity. Int. J. Environ. Res. Public Health.

[36] Eisenbeis CF, Lesinski GB, Anghelina M (2005). Phase I study of the sequential combination of interleukin-12 and interferon alfa-2b in advanced cancer: Evidence for modulation of interferon signaling pathways by interleukin-12. J. Clin. Oncol.: Off. J. Am. Soc. Clin. Oncol..

[37] Antonio Chiocca E, Yu JS, Lukas RV (2019). Regulatable interleukin-12 gene therapy in patients with recurrent high-grade glioma: Results of a phase 1 trial. Sci. Transl. Med..

[38] Miwa S, Nishida H, Tanzawa Y (2017). Phase 1/2 study of immunotherapy with dendritic cells pulsed with autologous tumor lysate in patients with refractory bone and soft tissue sarcoma. Cancer.

