# Supplementary material for: Influence of higher body mass index on postoperative nausea and vomiting in patients following thoracic surgery for lung cancer: a propensity score-matched cohort study
Source: Sci Rep. 2024 Jun 16;14:13873. doi: 10.1038/s41598-024-64686-1 (PMC11180656; doi:10.1038/s41598-024-64686-1)
Supplement: Supplementary file 1 — Supplementary Information. [file 41598_2024_64686_MOESM1_ESM.docx]

Supplementary table 1. Intraoperative anaesthetic data

| Agents | BMI: 18.5–25 kg/m^2^ | BMI≥25 kg/m^2^ | P-value |
| --- | --- | --- | --- |
| Midazolam dose (mg) | 5±0 | 5±0 | 1 |
| Rocuronium dose (mg) | 67.33±22.84 | 71.78±29.04 | 0.428 |
| Propofol dose (mg) | 345.27±170.29 | 340.88±180.90 | 0.899 |
| Sufentanil dose (µg) | 60.96±20.63 | 55.78±15.82 | 0.157 |
| Remifentanil dose (µg) | 494.08±343.18 | 535.22±436.09 | 0.607 |

^a^Continuous variables are presented as mean±standard deviation.

Abbreviations: BMI, body mass index


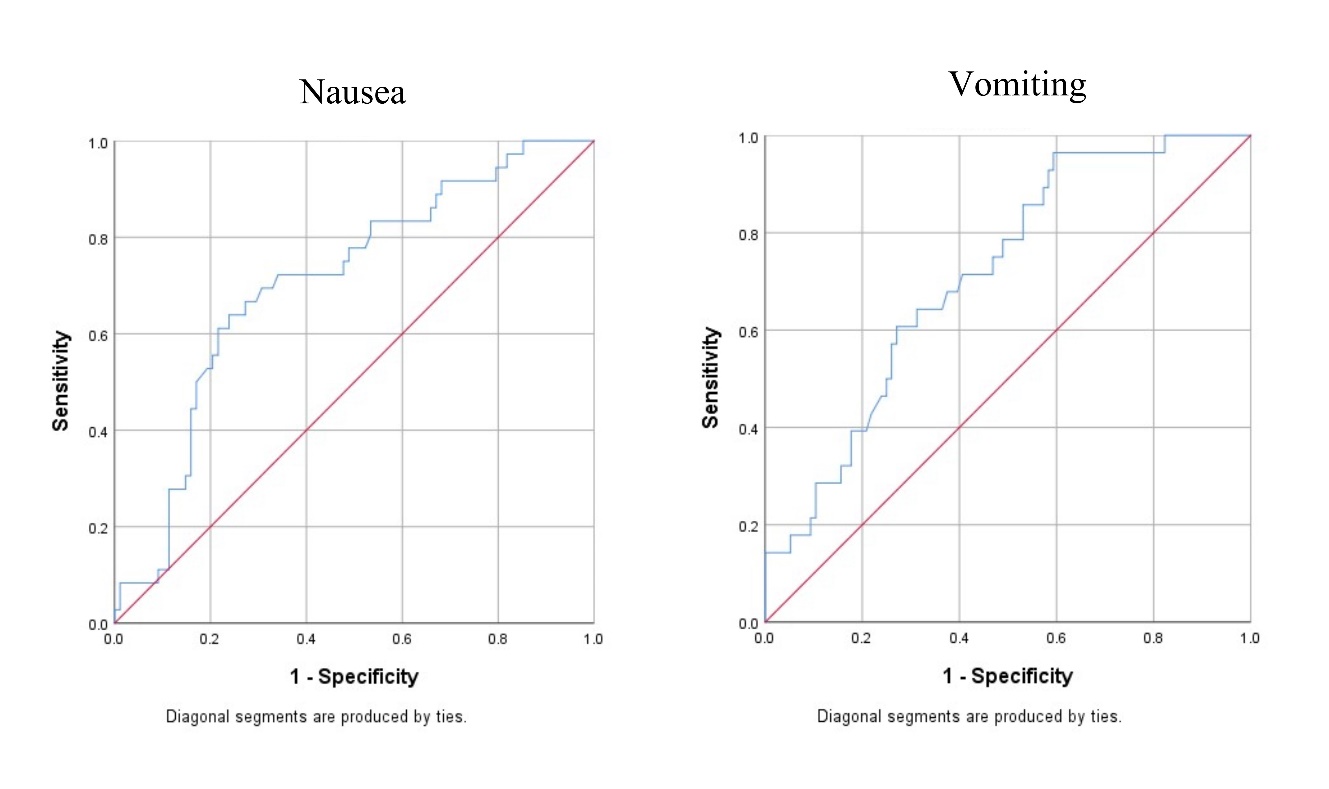


Supplementary Figure 1. Receiver operating characteristic (ROC) curve analysis for BMI and smoking history to predict nausea and vomiting.

Abbreviations: BMI, body mass index;


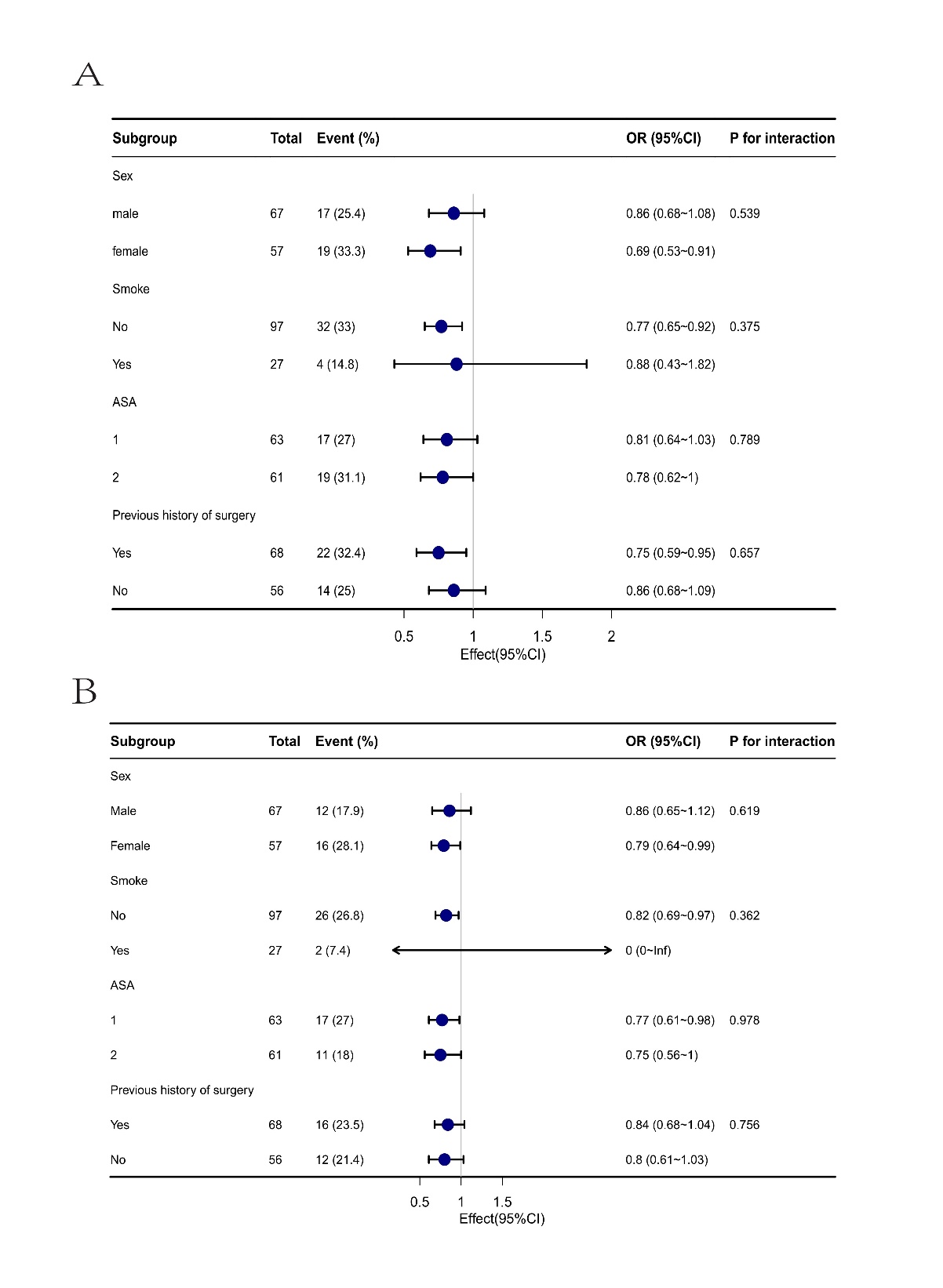


Supplementary Figure 2. Subgroup analysis. Subgroup analysis investigating the effect of BMI on nausea (A) and vomiting (B) at postoperative 72 h, according to sex (female vs male), history of smoking (yes vs no); ASA (1 vs 2) and history of surgery (yes vs no).

Adjusted for age, aCCI, duration of anaesthesia and resection mode.

Abbreviations: BMI, body mass index; SD, standard deviation; CI, confidence interval.
